# Splenic Volume Changes After Left Nephrectomy: A Retrospective Computed Tomography-Based Morphometric Study

**DOI:** 10.3390/diagnostics16193226

**Published:** 2026-10-06

**Authors:** Süniye Bilgiç Sal, Keziban Karacan, Alper Karacan, Mehtap Erdoğan

**Affiliations:** 1 Department of Anatomy, Faculty of Medicine, Kocaeli University, 41380 Kocaeli, Türkiye; suniyebilgic@gmail.com; 2 Department of Anatomy, Faculty of Medicine, Sakarya University, 54290 Sakarya, Türkiye; kkaracan@sakarya.edu.tr; 3 Department of Radiology, Faculty of Medicine, Sakarya University, 54290 Sakarya, Türkiye; alperkaracan@sakarya.edu.tr

**Keywords:** spleen, spleen volume, left nephrectomy, computed tomography, morphometry, volumetric change

## Abstract

**Background/Objective**: Spleen volume can vary depending on physiological conditions, systemic diseases, and various clinical factors. However, the relationship between the anatomical changes resulting from the surgical removal of an organ adjacent to the spleen and spleen volume has not been sufficiently studied. The aim of this study was to evaluate changes in spleen volume following left nephrectomy and to investigate the associations of sex and the time interval between preoperative and postoperative computed tomography (CT) examinations with these changes. **Methods**: In this retrospective study, preoperative and postoperative abdominal CT images of 62 patients (30 women, 32 men; age range 37–85 years) who underwent left radical nephrectomy were evaluated. Spleen volume was calculated by manually delineating the spleen borders on axial CT slices and using the TeraRecon imaging system. Preoperative and postoperative spleen volumes were compared; furthermore, the relationship between volume change and sex, as well as the time interval between the two CT scans, was evaluated. **Results**: The mean spleen volume was 238.41 ± 80.99 cm^3^ before surgery and was found to be 259.81 ± 104.40 cm^3^ after surgery. An average increase of 9.0% in spleen volume was observed following nephrectomy, and this increase was statistically significant (*p* = 0.005). Although spleen volume increased in both women and men, the change in volume did not show a significant difference based on sex (14.07 ± 53.69 cm^3^ in women; 28.28 ± 62.32 cm^3^ in men; *p* = 0.339). No statistically significant difference in splenic volume change was detected between the predefined CT-interval groups (*p* = 0.633). Postoperative imaging time was not significantly correlated with splenic volume change when analyzed continuously (Spearman’s ρ = −0.128, *p* = 0.320). **Conclusions**: Mean CT-derived splenic volume was higher on postoperative than on preoperative examinations. However, because the study lacked a control group and longitudinal data on relevant clinical confounders, this difference cannot be attributed specifically to left nephrectomy or interpreted as definitive evidence of true splenic hypertrophy.

## 1. Introduction

The spleen is one of the largest lymphoid organs in the human body and performs important functions such as supporting the immune system, maintaining hematological homeostasis, and storing blood. Located in the upper left quadrant of the abdominal cavity, the spleen is in close anatomical proximity to the stomach, pancreas, diaphragm, and, in particular, the left kidney [1,2,3,4]. The spleen’s anatomical location and morphological characteristics are important for the evaluation of both normal anatomical variations and various physiological and pathological conditions.

It is known that spleen volume can vary significantly among individuals and can be influenced by age, body weight, sex, nutritional status, physical activity, infections, inflammatory diseases, and various systemic diseases [5,6,7,8]. Changes in spleen volume have been investigated in the literature in various clinical conditions, such as chronic kidney disease, hemodialysis, liver diseases, inflammatory bowel diseases, acute stroke, and abdominal trauma [9,10,11,12,13,14]. These studies demonstrate that the spleen’s volumetric properties represent a dynamic structure capable of changing in response to physiological and pathological changes occurring in the body.

Computed tomography (CT) is one of the imaging methods commonly used to assess spleen morphology and volume. The evaluation of cross-sectional areas derived from CT images and the use of three-dimensional volumetric measurement methods enable a more objective and sensitive determination of spleen volume [15,16]. For this reason, CT-based volumetric measurements are used as an important method in the evaluation of the spleen’s morphometric characteristics.

Because the spleen and left kidney are anatomically adjacent, changes in splenic position or configuration after left nephrectomy are conceivable, but have not been established. Nephrectomy is a surgical procedure used in the treatment of various kidney diseases and involves complete or partial removal of the kidney [17]. Although relationships between kidney disease, renal replacement therapy, and spleen volume have been investigated, few studies have compared CT-derived splenic volume before and after left nephrectomy [9,10]. Any anatomical mechanism linking kidney removal to a change in splenic volume remains untested.

In the current literature, changes in spleen volume have mostly been evaluated in the context of physiological processes, systemic diseases, or trauma. Few studies have compared preoperative and postoperative CT-derived splenic volumes within the same patients undergoing left nephrectomy. Accordingly, postoperative differences in CT-derived splenic volume warrant descriptive investigation but should not be presumed to represent true tissue hypertrophy or a mechanical effect of the space created after nephrectomy.

The aim of this study was to compare CT-derived splenic volume before and after left nephrectomy and to examine whether splenic volume change was associated with sex or the interval between preoperative and postoperative CT examinations. We hypothesized that CT-derived splenic volume would differ between the preoperative and postoperative examinations.

## 2. Materials and Methods

### 2.1. Study Design and Patient Population

In this retrospective, observational study, all consecutive patients who underwent left radical nephrectomy between February 2013 and September 2023 were screened. Surgical records and all available CT examinations were reviewed. Availability of paired preoperative and postoperative abdominal CT examinations suitable for splenic volumetry was required for inclusion. A total of 145 patients were assessed for eligibility. Eighty-three patients were excluded: preoperative CT only (*n* = 25), postoperative CT only (*n* = 17), inaccessible preoperative CT with postoperative CT available (*n* = 8), unavailable or inaccessible CT examinations (*n* = 15), thoracic CT only or absence of a complete abdominal CT pair (*n* = 3), splenectomy (*n* = 4), right nephrectomy or discordant surgical history (*n* = 3), exclusion reason not recorded (*n* = 7), and uncertain eligibility (*n* = 1). The final analysis included 62 patients with paired preoperative and postoperative CT examinations. The included cohort consisted of patients undergoing left radical nephrectomy; simple nephrectomy, partial nephrectomy, and nephroureterectomy were not included. The study was conducted in accordance with the Declaration of Helsinki and was approved by the Sakarya University Faculty of Medicine Non-Interventional Ethics Committee (Approval No. 332; Date: 31 October 2023).

**Figure 1:**
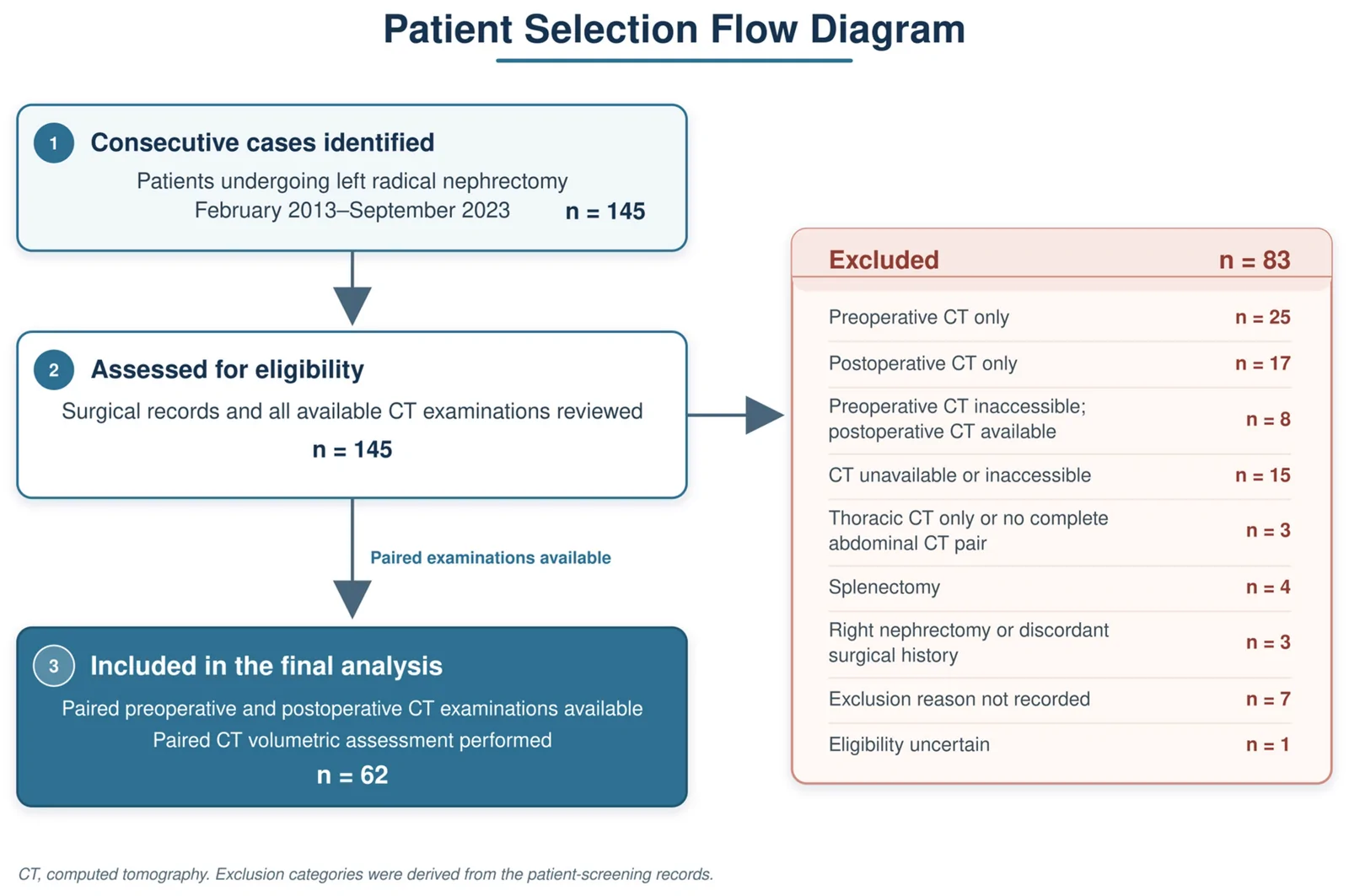
Flow diagram of patient selection.

The final study cohort consisted of 62 patients. Thirty (48.4%) were women, and 32 (51.6%) were men. The patients’ ages ranged from 37 to 85 years, with a mean age of 63.42 ± 10.83 years. The mean age of female patients was 65.13 ± 10.04 years (range, 44–85 years), while the mean age of male patients was 61.81 ± 11.45 years (range, 37–83 years). The patient-selection process is summarized in Figure 1.

In accordance with hospital protocol, abdominal CT scans were performed on an empty stomach. The aim was to minimize the potential impact on the measurements of changes in spleen volume that might occur during the postprandial period.

### 2.2. Computed Tomography Protocol

Abdominal CT scans of the patients were performed in the supine position and in the axial plane. Images were acquired using a 64-slice multidetector CT scanner (Aquilion 64, Toshiba Medical Systems, Tokyo, Japan). The scanning protocol included a tube voltage of 120 kV, an automatic tube current of 120–380 mA, a slice thickness of 5 mm, and a slice spacing of 5 mm. The gantry rotation time was 0.5 s, and the helical pitch was 1.0875:1 or 1.375:1.

Preoperative and postoperative images had to be suitable for evaluating the morphology and borders of the spleen in both periods. Patients without preoperative or postoperative abdominal CT scans were excluded from the study.

### 2.3. Measurement of Spleen Volume

Spleen volume was measured at the Department of Radiology, Sakarya University Training and Research Hospital, using TeraRecon Aquarius iNtuition software, version 4.4.13. All volumetric measurements were performed by a single radiologist with 15 years of professional experience. The examinations were presented in random order with examination dates and clinical identifiers concealed. Complete blinding to operative status was not feasible because the absence of the left kidney was visible on postoperative images.

**Figure 2:**
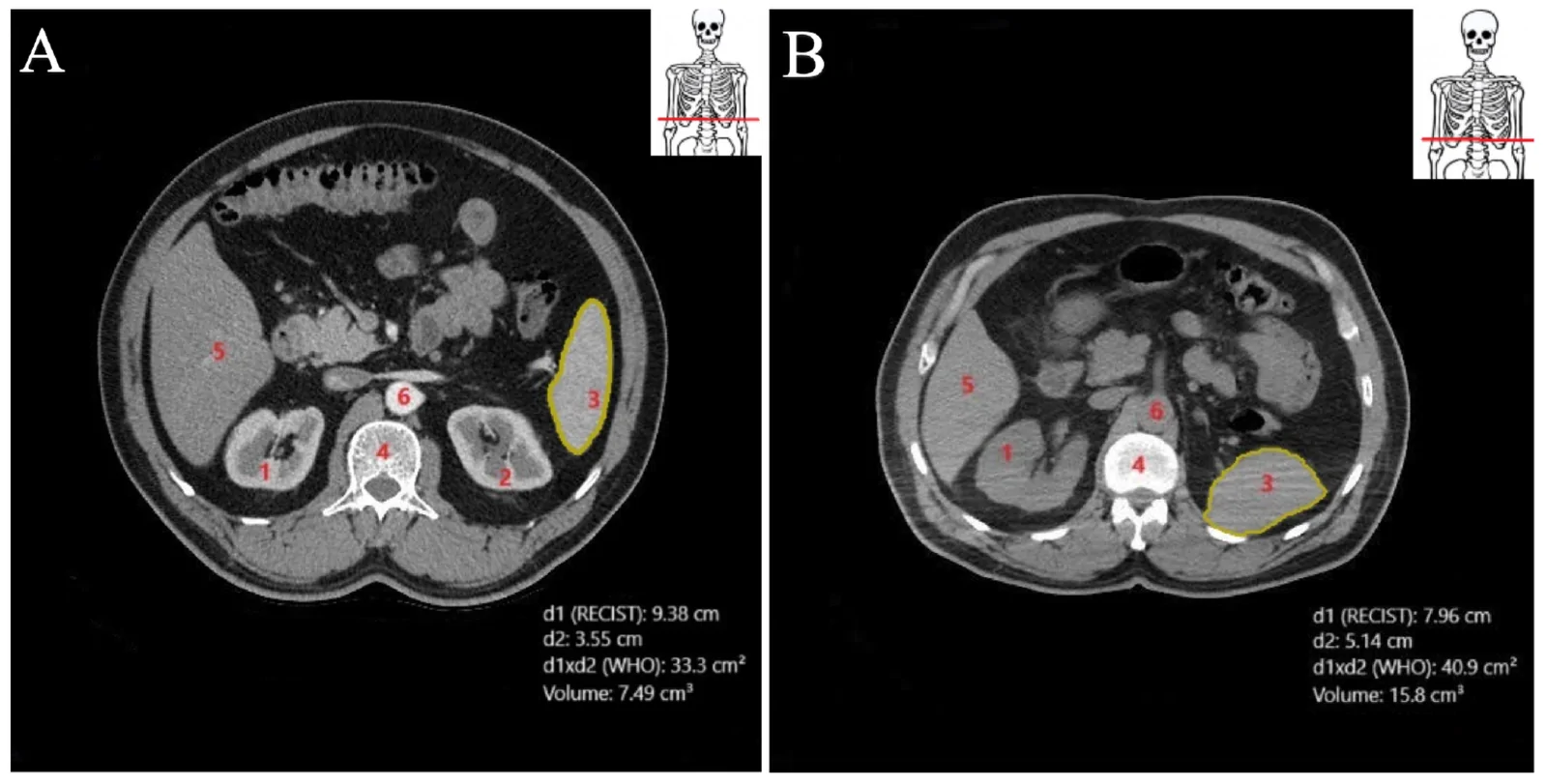
Representative axial CT images from the same patient obtained (**A**) 51 days before left nephrectomy and (**B**) 257 days after surgery; the interval between the two CT examinations was 308 days. The panels were selected separately as representative sections and do not constitute anatomically matched single-slice images. Both panels are shown in axial orientation. The yellow contours delineate the spleen. Numbered labels identify (1) the right kidney, (2) the left kidney (present only in panel (**A**)), (3) the spleen, (4) a lumbar vertebral body, (5) the liver, and (6) the abdominal aorta. The whole-organ segmented splenic volumes were 167.2 cm^3^ in panel (**A**) and 210.0 cm^3^ in panel (**B**). In panel (**A**), the displayed splenic cross-sectional measurements were d1 = 9.38 cm, d2 = 3.55 cm, d1 × d2 = 33.3 cm^2^, and volume = 7.49 cm^3^. In panel (**B**), the corresponding measurements were d1 = 7.96 cm, d2 = 5.14 cm, d1 × d2 = 40.9 cm^2^, and volume = 15.8 cm^3^.

The spleen borders were manually delineated on axial CT slices. The cross-sectional area on each slice was multiplied by the corresponding slice thickness, and the resulting volumes were summed to obtain the total spleen volume. To reduce measurement variability associated with manual segmentation, the same radiologist repeated each splenic volume measurement three times during a single assessment session. The mean of the three measurements was used for analysis. Intraobserver reliability across the three repeated measurements was assessed using the intraclass correlation coefficient (ICC = 0.96; 95% CI: 0.94–0.97), indicating excellent repeatability. Spleen volumes were calculated separately for preoperative and postoperative images using the same measurement method. Representative axial CT images demonstrating manual delineation of the spleen in the same patient are presented in Figure 2A (preoperative) and Figure 2B (postoperative).

### 2.4. Evaluation of the Time Interval Between Preoperative and Postoperative Images

To evaluate the relationship between the time interval between preoperative and postoperative CT scans and changes in spleen volume, the patients were divided into two groups. The first group consisted of 32 patients for whom the time interval between preoperative and postoperative CT scans was 0–18 months; the second group consisted of 30 patients for whom this interval was 19 months or longer. The 18-month cutoff was used for an exploratory comparison of relatively earlier and later imaging intervals and was not based on an established clinical threshold. Preoperative and postoperative spleen volumes and volume changes were compared in both groups.

### 2.5. Statistical Analysis

Statistical analyses were performed using the Statistical Package for the Social Sciences (SPSS), version 26.0 (IBM Corp., Armonk, NY, USA). The normality of continuous variables was assessed using the Shapiro–Wilk and Kolmogorov–Smirnov tests. In evaluating the assumption of normal distribution, both the test results and the distribution characteristics were taken into account.

A paired-samples *t*-test was used to compare preoperative and postoperative spleen volumes. An independent-samples *t*-test was used to assess whether changes in spleen volume differed by sex and between the predefined CT-interval groups. In all statistical analyses, a *p*-value of <0.05 was considered statistically significant.

The intervals from preoperative CT to surgery and from surgery to postoperative CT were calculated separately in days. Because these intervals showed skewed distributions, they were summarized using the median, interquartile range (IQR), and minimum–maximum values. The association between the interval from surgery to postoperative CT, analyzed as a continuous variable, and the change in splenic volume was evaluated using Spearman’s rank correlation coefficient.

## 3. Results

### 3.1. Demographic Characteristics of the Study Group

A total of 62 patients were included in the study. Thirty (48.4%) of the patients were female, and 32 (51.6%) were male. The mean age of the patients was 63.42 ± 10.83 years, with an age range of 37–85 years. The mean age of female patients was 65.13 ± 10.04 years (range: 44–85 years), while the mean age of male patients was 61.81 ± 11.45 years (range: 37–83 years). The demographic characteristics of the study group are presented in Table 1.

### 3.2. Comparison of Preoperative and Postoperative Spleen Volumes

The mean preoperative spleen volume was 238.41 ± 80.99 cm^3^ (94.40–416.50 cm^3^), while the mean postoperative spleen volume was found to be 259.81 ± 104.40 cm^3^ (86.50–508.80 cm^3^). An average increase of 21.40 ± 58.27 cm^3^ in spleen volume was observed postoperatively. The difference between preoperative and postoperative spleen volumes was statistically significant (*p* = 0.005). Data regarding preoperative and postoperative spleen volumes are presented in Table 2.

### 3.3. Evaluation of Spleen Volume Changes by Sex

Preoperative spleen volume did not show a statistically significant difference between female and male participants (*p* = 0.399). Similarly, no statistically significant difference in postoperative spleen volume was detected by sex (*p* = 0.233). The distribution of splenic volumes by sex is presented in Table 3.

The mean change in splenic volume was 14.07 ± 53.69 cm^3^ in women and 28.28 ± 62.32 cm^3^ in men. No statistically significant difference in splenic volume change was detected between the sex groups (*p* = 0.339). Splenic volume change by sex is shown in Table 4.

### 3.4. Association Between CT Timing and Change in Splenic Volume

The median interval from preoperative CT to surgery was 39 days (IQR, 16–67 days; range, 0–2116 days). The median interval from surgery to postoperative CT was 485 days (IQR, 215.5–1012.5 days; range, 33–2689 days). The median overall interval between the preoperative and postoperative CT examinations was 592 days (IQR, 278.25–1109.75 days; range, 44–2985 days). When the interval from surgery to postoperative CT was analyzed as a continuous variable, no statistically significant correlation with splenic volume change was detected (Spearman’s ρ = −0.128, *p* = 0.320).

For the exploratory comparison of relatively earlier and later imaging intervals, patients were divided into two predefined groups: 0–18 months and ≥19 months. The first group consisted of 32 patients, and the second group consisted of 30 patients.

The mean preoperative spleen volume was 237.73 ± 90.58 cm^3^ in the 0–18-month group and 239.13 ± 70.88 cm^3^ in the ≥19-month group. Postoperative mean spleen volumes were found to be 262.58 ± 116.11 cm^3^ and 256.86 ± 92.17 cm^3^, respectively. The mean increase in spleen volume was 24.85 ± 62.37 cm^3^ in the 0–18-month group and 17.72 ± 54.37 cm^3^ in the ≥19-month group.

Although an increase in spleen volume was observed in both groups after surgery, no statistically significant difference was found in spleen volume change between the 0–18-month and ≥19-month groups (*p* = 0.633). The mean increase in spleen volume for the entire study group was 21.40 ± 58.27 cm^3^. Data on changes in spleen volume according to the time interval between CT scans are presented in Table 5.

## 4. Discussion

Spleen volume can be influenced by many physiological and pathological factors, such as age, sex, body weight, nutritional status, physical activity, and various diseases. Therefore, these variables should be considered when evaluating spleen morphometry. In the present study, mean CT-derived splenic volume was higher on postoperative than on preoperative examinations. No statistically significant difference in splenic volume change was detected between the examined sex groups or between the predefined CT-interval groups. In addition, no statistically significant correlation was detected between postoperative imaging time and splenic volume change when postoperative time was analyzed continuously.

In our study, the mean pre-nephrectomy spleen volume was found to be 238.41 ± 80.99 cm^3^. This value is quite close to the 236.89 ± 77.58 mL reported by Linguraru et al. in healthy individuals [9]. This value is broadly comparable to the spleen volumes reported by Linguraru et al. in their reference population. The increase in the mean spleen volume to 259.81 ± 104.40 cm^3^ after nephrectomy, which was found to be statistically significant (*p* = 0.005), indicates that changes in spleen morphometry may occur following the removal of the left kidney.

Previous studies have shown that spleen volume is related to age and body build. Ünlü and Ilgar reported that spleen volume increases as children get older [18]. Schlesinger and colleagues, on the other hand, noted that spleen volume is more strongly correlated with body weight than with age [19]. However, our study consists of a group predominantly composed of adults and the elderly, and the primary comparison was made using pre- and post-nephrectomy imaging of the same patients. Therefore, it is believed that the effect of interindividual anatomical differences has been partially reduced. Lo et al. examined splenic volume changes after radical nephroureterectomy in patients with upper tract urothelial carcinoma, primarily to evaluate their prognostic significance [17]. Their study provides clinical context for postoperative splenic volume changes in a different patient population, but it does not establish that the anatomical space created by kidney removal mechanically causes splenic enlargement.

The mechanism underlying the observed postoperative difference in CT-derived splenic volume remains uncertain. One possible, but untested, explanation is that left nephrectomy alters the spatial relationships and position of the adjacent spleen. The present findings do not demonstrate that the anatomical space created by kidney removal causes true splenic enlargement. However, a hemodynamic contribution cannot be excluded. Yoshida et al. emphasized the close relationship between splenic hemodynamics, portal circulation, and splenic congestion, suggesting that alterations in venous hemodynamics may contribute to changes in spleen volume [20]. More recently, Chen et al. demonstrated a significant association between CT-derived spleen volume and hepatic venous pressure gradient, further supporting the relationship between spleen volume and portal hemodynamic alterations [21]. Although ligation of the left renal artery and vein during nephrectomy alters the regional vascular anatomy, there is currently insufficient evidence to establish that these changes directly cause increased splenic blood flow or venous congestion. Accordingly, the postoperative difference in CT-derived splenic volume could reflect postoperative anatomical adaptation, hemodynamic changes, or a combination of these mechanisms. Because our study was based on retrospective CT volumetry and did not include direct measurements of splenic or portal blood flow or venous pressure, the relative contribution of these mechanisms cannot be determined. Further prospective studies incorporating Doppler ultrasonography or other vascular imaging techniques are warranted to clarify the underlying mechanism.

An untested anatomical explanation for the observed postoperative difference in CT-derived splenic volume should also be considered. Following left nephrectomy, the space created within the left renal fossa may alter the spatial relationships of adjacent organs. Downward displacement or rotation of the spleen could change its configuration across axial CT sections and potentially influence CT-based volumetric estimates without necessarily representing true enlargement or hypertrophy of the splenic parenchyma. Because this study relied on volumetric segmentation rather than direct assessment of splenic position, orientation, or parenchymal thickness, it cannot determine whether the observed difference represents true tissue enlargement, positional remodeling, measurement effects, or a combination of these factors. Future studies incorporating quantitative assessment of splenic position, orientation, and shape, preferably using three-dimensional imaging techniques, are warranted to distinguish these possibilities.

Previous studies examining the relationship between the kidneys and the spleen also suggest that there may be a correlation between the morphometric characteristics of these two organs. Araújo and Suassuna reported that spleen volume was higher in patients with chronic kidney disease compared to a control group and attributed this finding to chronic inflammation [22]. Similarly, Platts and colleagues demonstrated the effects of chronic kidney disease and hemodialysis on spleen size [23]. However, these studies addressed kidney disease or changes in kidney function from a pathophysiological perspective. The present study identifies a postoperative difference in CT-derived splenic volume but does not establish its mechanism or causal attribution to nephrectomy.

In our study, the increase in spleen volume following nephrectomy was found to be 14.07 ± 53.69 cm^3^ in women and 28.28 ± 62.32 cm^3^ in men; however, the difference between the sexes was not statistically significant (*p* = 0.339). When preoperative and postoperative spleen volumes were evaluated separately, no significant difference was found between the sexes. The between-sex difference in splenic volume change was not statistically significant (*p* = 0.339); this finding does not exclude a sex-related difference.

Since spleen volume can also be influenced by nutritional status, the fact that the imaging in our study was performed in a fasting state is a significant advantage. Garnitschnig et al. reported a marked and transient increase in spleen volume following a meal [24]. The fact that the imaging in our study was performed in a fasting state, as required by hospital protocol, may have reduced the variability that this factor could have introduced.

In our study, the time interval between preoperative and postoperative imaging was also evaluated using predefined groups of 0–18 months and 19 months or longer. No statistically significant difference in splenic volume change was detected between these groups (*p* = 0.633). When the interval from surgery to postoperative CT was analyzed as a continuous variable, no statistically significant correlation was detected between postoperative imaging time and splenic volume change (Spearman’s ρ = −0.128, *p* = 0.320). These findings should not be interpreted as evidence that splenic volume change was independent of follow-up duration.

### Limitations

The main limitations of this study are its retrospective design, relatively small sample size, and absence of a right-nephrectomy or non-nephrectomized control group undergoing serial CT. Although paired measurements reduce between-patient variability, they cannot distinguish a change specifically related to left nephrectomy from normal biological or temporal variation. Body size or BMI, weight change, renal function, liver disease, portal hypertension, malignancy status and treatment, infection or inflammation, and hydration or fluid status were not available for reliable assessment at both imaging time points. Consequently, these potential time-varying influences could not be included in an adjusted analysis. The observed difference in CT-derived splenic volume should therefore be regarded as a descriptive, hypothesis-generating finding and cannot be attributed specifically to left nephrectomy. The within-patient design and repeated volumetric measurements by the same experienced radiologist are strengths of the study, but they do not eliminate these limitations.

The use of 5 mm CT sections with 5 mm intervals for manual splenic segmentation is an important limitation. Thin-section reconstructions were not obtained in this retrospective cohort and were unavailable for repeat volumetry. Partial-volume effects at the splenic margins may have affected volume estimates, but the magnitude and direction of this error cannot be quantified from the available images. Therefore, the measured difference in CT-derived splenic volume should not be regarded as unequivocal evidence of true splenic enlargement.

## 5. Conclusions

In this retrospective within-patient analysis, mean CT-derived splenic volume was higher on postoperative than on preoperative examinations. No statistically significant difference in splenic volume change was detected between the examined sex groups or between the predefined CT-interval groups, and no statistically significant correlation was detected between postoperative imaging time and splenic volume change when postoperative time was analyzed continuously. Because the study lacked a control group and longitudinal data on relevant clinical confounders, the observed difference cannot be attributed specifically to left nephrectomy or interpreted as definitive evidence of true splenic hypertrophy. Controlled prospective studies using thin-section CT volumetry are required to confirm this observation.

## Figures and Tables

**Table 1:** Demographic characteristics of the study population.

Characteristic	Total (*n* = 62)	Female (*n* = 30)	Male (*n* = 32)
Age (years), mean ± SD	63.42 ± 10.83	65.13 ± 10.04	61.81 ± 11.45
Age range (years)	37–85	44–85	37–83
Sex, *n* (%)	62 (100)	30 (48.4)	32 (51.6)

**Table 2:** Comparison of preoperative and postoperative splenic volume.

Splenic Volume	*n*	Mean ± SD (cm^3^)	Range (cm^3^)	*p*-Value
Preoperative	62	238.41 ± 80.99	94.40–416.50	0.005
Postoperative	62	259.81 ± 104.40	86.50–508.80	

**Table 3:** Preoperative and postoperative splenic volumes according to sex.

Sex	*n*	Preoperative Volume, Mean ± SD (cm^3^)	Postoperative Volume, Mean ± SD (cm^3^)
Female	30	229.39 ± 77.03	243.45 ± 96.47
Male	32	246.87 ± 84.87	275.14 ± 110.63
*p*-value		0.399	0.233

*p*-values represent comparisons between female and male patients.

**Table 4:** Change in splenic volume after left nephrectomy according to sex.

Sex	*n*	Change in Splenic Volume, Mean ± SD (cm^3^)
Female	30	14.07 ± 53.69
Male	32	28.28 ± 62.32
Total	62	21.40 ± 58.27

*p* = 0.339 for comparison of spleen volume change between female and male patients.

**Table 5:** Comparison of splenic volume change according to the interval between preoperative and postoperative CT examinations.

CT Interval	*n*	Preoperative Volume, Mean ± SD (cm^3^)	Postoperative Volume, Mean ± SD (cm^3^)	Change, Mean ± SD (cm^3^)
0–18 months	32	237.73 ± 90.58	262.58 ± 116.11	24.85 ± 62.37
≥19 months	30	239.13 ± 70.88	256.86 ± 92.17	17.72 ± 54.37
Total	62	238.41 ± 80.99	259.81 ± 104.40	21.40 ± 58.27

*p* = 0.633 for comparison of spleen volume change between the two CT interval groups.

## Data Availability

The original contributions presented in this study are included in the article. Further inquiries can be directed to the corresponding author.

## Abbreviations

The following abbreviations are used in this manuscript:

CT	Computed tomography
ICC	Intraclass correlation coefficient
SD	Standard deviation
SPSS	Statistical Package for the Social Sciences
